# Supplementary material for: A latent class analysis of the socio-demographic factors and associations with mental and behavioral disorders among Australian children and adolescents
Source: PLoS One. 2023 May 18;18(5):e0285940. doi: 10.1371/journal.pone.0285940 (PMC10194994; doi:10.1371/journal.pone.0285940)
Supplement: S1 Table — (DOCX) [file pone.0285940.s001.docx]

**Table S1:** Association of socio-demographic characteristics with mental and/or behavioral disorders of children and adolescents

| Socio-demographic Characteristics | | | Mental and/or behavioral disorders ^a^ | | P-value |
| --- | --- | --- | --- | --- | --- |
|  |  |  | Yes (n)  (unweighted) | %  (weighted) |  |
| Individual-level | Age  (mean ± SD:14.6 ± 2.04) | 11 to <15 years | 548 | 47.9 | 0.05 |
|  |  | 15 to <18 years | 664 | 52.1 |  |
|  | Sex | Male | 638 | 53.4 | <0.001 |
|  |  | Female | 574 | 46.6 |  |
| Household-level | Regional status ^b^ | Metropolitan | 739 | 60.5 | <0.001 |
|  |  | Non-metropolitan | 473 | 39.5 |  |
|  | Family blending ^c^ | Intact family | 670 | 52.8 | <0.001 |
|  |  | Other families | 542 | 47.2 |  |
|  | Both parents live in the household | Yes | 716 | 57.2 | <0.001 |
|  |  | No | 496 | 42.8 |  |
| Socio-economic level | Household income/year | Low | 359 | 32.5 | <0.001 |
|  |  | Middle | 538 | 42.3 |  |
|  |  | High | 315 | 25.2 |  |
|  | Parental education | Bachelor | 340 | 27.7 | <0.001 |
|  |  | Diploma | 460 | 38.0 |  |
|  |  | Year 12/below | 412 | 34.3 |  |
|  | Parental employment | Employed | 862 | 70.9 | <0.001 |
|  |  | Unemployed | 350 | 29.1 |  |
|  | IRSAD quintile ^d^ | Lowest | 236 | 19.5 | <0.001 |
|  |  | Second | 226 | 19.4 |  |
|  |  | Third | 239 | 20.1 |  |
|  |  | Fourth | 247 | 20.4 |  |
|  |  | Highest | 264 | 20.6 |  |
| n= unweighted number of respondents; % =weighted percentage | | | | | |
