# Supplementary material for: A latent class analysis of the socio-demographic factors and associations with mental and behavioral disorders among Australian children and adolescents
Source: PLoS One. 2023 May 18;18(5):e0285940. doi: 10.1371/journal.pone.0285940 (PMC10194994; doi:10.1371/journal.pone.0285940)
Supplement: Attachment — Submitted filename: Comments on the paper.docx [file pone.0285940.s002.docx]

Comments on the paper

General:

Thank you for giving me the opportunity to read your interesting manuscript.

Overall, I find the manuscript objective and the methodological approach very interesting. From my point of view, however, the manuscript needs a major revision. In particular, the operationalization of "mental health" should be in accordance with the standards.

It would have been easier to comment on the paper if page and line numbers had been added to the text.

Please proofread your manuscript meticulously and correct the spelling and grammatical mistakes. In parts the wording lacks accuracy

Abstract:

Please include the year of data collection in the methods section of the abstract.

Background:

In the section of the background chapter that begins with “Therefore, instead of analyzing each socio-demographic factor separately, in this study, we have considered a latent class analysis (LCA)…”, I would describe the research gap, not the objective of the study. For example, you could write: Up to date, in these studies, socioeconomic and sociodemographic factors were only analyzed separately, not in combination.

In the last paragraph of the background I would recommend to write:

“Therefore, the FIRST objective of this study is to identify the cluster …”

AND:

“The SECOND objective of this study is to explore the link between adolescent mental health with class membership and adjusted controlling factors.”

Method:

Please delete the year in brackets of reference 22.

In the chapter “Measurements” please describe the measurement and operationalization of the sociodemographic variables. The description of the social hierarchy of Australia and the “Diagram of Australian social hierarchy and its‘ characteristics” should be presented in the background chapter, not in the methods chapter. Please only provide information related to your study methodology.

Please explain the income variable in more detail. Is the income of the household used? Was the number of household members taken into account (household equivalent income? Is it gross or net income)?

What is the difference between “family blending (intact family vs other families)” and “parents’ living status (whether both parents live in the household or not)”? Is it necessary to use both indicators?

I suppose that the two variables measure almost the same thing. Do the variables refer to the biological parents?

I would suggest to use the variable “family type” (“couple family” versus “one-parent family”) as Lawrence et al. (2016) (doi:10.1177/0004867415617836) does. Or alternatively: “original family (yes/no)”.

Furthermore, I am of the opinion that both variables measure family structure and not family functioning. Although it is known that there is a strong association between family structure and family functioning, they are not the same. Therefore, please move the sentence “In 2015, a study found that adolescents from non-intact households had a lower perception of family functioning, father‘s and mother‘s behavioral control, paternal psychological control, and parent-child relational traits than adolescents from intact households [25].” into the discussion.

I would recommend to move the first paragraph of the chapter “Study participants and data analysis” to the end of the chapter “Data source and study design” and rename the chapter "Statistical Analysis".

I don’t understand why you adjusted for the variables “inability to pay gas, electricity, or telephone bills on time”, “could not pay the mortgage or rent on time”, “adults/children have gone without meals”, and “households receive a carer benefit or pension in relation to child”. I think it is an overadjustment. You may want to analyze whether the association between class membership and mental disorder is mediated by these variables. But that is not the subject of your study. I recommend to adjust only for household size.

Were weights and survey procedures used in the statistical analysis? Due to the sampling (complex structure of sample), this should be done.

Results:

For the labeling of the age groups, a wrong mathematical symbol was used. It must be “≥15”. Alternatively, you could write “11 to <15” and “15 to <18” (see line 2 of the results chapter and Table 1).

“Moreover, the study results showed that 68.1% of parents live in households.” What does this mean?

You reported a 12month prevalence of 63.5 % for mental disorders in adolescents!

In the manuscript of Lawrence et al. (2016) (doi:10.1177/0004867415617836), who used the same data, the 12month prevalence of mental disorders was 13.9 %. I recommend strongly to check the operationalization of the variable. For reasons of comparability, please build the variable analogously to the mentioned reference paper.

The associations between the single sociodemographic variables and mental disorders are not the subject of the study. Therefore, I would delete the section “Distribution of socio-demographic characteristics with adolescents’ mental health” and move Table 2 into the supplement.

After the description of the study sample, I recommend to start with the classes you found.

The description of the clusters could be more concise. Please highlight the main characteristics of each class.

The cluster labels of class 3 “Mobile middle class” and class 4 “Growing wealthy class” cannot be deduced from the variables used in the LCA. For example, the most striking criterion of the "growing wealthy class" is that the adolescent does not live with both parents. I recommend to give the classes labels that result from the associations reported in Table 3.

In Table 4, I suggest to use the recently introduced labels for the classes and to put the class numbers in brackets.

Following the description of the classes, I would like to know the prevalences for mental disorders stratified by class. That is the main result of your study!

In the section “Regression modeling” I would start with the sentence “Table 5 presents the results of binary logistic regressions …”. The sentences before belong into the method chapter.

Regarding Table 5, 2 decimals suffice for reporting the OR and the 95 % CI.

The calculation of the OR only allows comparisons to the reference group. If you would calculate predictive margins, you could compare all classes with each other.

Furthermore, when the prevalence of the disease is high, the Prevalence Ratio (PR) would be the better estimator.

Discussion:

The aim of your study is to analyze the association between the sociodemographic classes and adolescents’ mental disorder and not to analyzed the associations between the single sociodemographic variables and mental disorder. In particular, the results for your approach should be reported and discussed. Please revise the first paragraph of the discussion accordingly.

The limitations of the study are missing completely.

Neither the discussion nor the conclusions point out the advantage of class analysis over bivariate correlation analysis.

References:

Please doublecheck the references. For some references the information is not complete (e.g. reference 24).
