# Supplementary material for: A latent class analysis of the socio-demographic factors and associations with mental and behavioral disorders among Australian children and adolescents
Source: PLoS One. 2023 May 18;18(5):e0285940. doi: 10.1371/journal.pone.0285940 (PMC10194994; doi:10.1371/journal.pone.0285940)
Supplement: Attachment — Submitted filename: Response to Reviewers.docx [file pone.0285940.s003.docx]

**Response to the academic editor and reviewers’ comments**

**Revised title of the manuscript:**

**“A latent class analysis of the socio-demographic factors and mental and behavioral disorders of Australian children and adolescents”**

| **Academic editor** | |
| --- | --- |
| Editor’s comments | Author’s response |
| After careful consideration, we feel that it has merit but does not fully meet PLOS ONE’s publication criteria as it currently stands. Therefore, we invite you to submit a revised version of the manuscript that addresses the points raised during the review process.  Please include the following items when submitting your revised manuscript:   - A rebuttal letter that responds to each point raised by the academic editor and reviewer(s). You should upload this letter as a separate file labeled 'Response to Reviewers'. - A marked-up copy of your manuscript that highlights changes made to the original version. You should upload this as a separate file labeled 'Revised Manuscript with Track Changes'. - An unmarked version of your revised paper without tracked changes. You should upload this as a separate file labeled 'Manuscript'. | Thank you so much for giving us the opportunity to submit a revised version of the manuscript.  We have addressed all the comments and hope the quality of this paper has now been improved substantially.  Please find attached our revised manuscript (a marked-up copy labelled ‘Revised Manuscript with Track Changes’ and an unmarked version labelled 'Manuscript') and below our point-by-point response to all reviewers. |

| Reviewers# 1 | | | |
| --- | --- | --- | --- |
| Sl. No. | Reviewer’s comments | Author’s response |  |
| Major points | |  |  |
| 1. | Study rationale. Why would it be important to use LCA to study SES?  In the abstract it reads that ” However, no study has yet been conducted on a model-based cluster analysis of socio-demographic characteristics with mental health.”, but why would be important to use certain statistical technique? | Thanks for asking these questions.  In this research, we did not use LCA to study socioeconomic status only. Rather we used it to identify the sociodemographic classes based on three different levels, namely individual level, household level, and socio-economic level. This classification is based on the landmark report of the ANUpoll 2015 which showed that Australian population can be divided into five distinct classes depending on their income and wealth, regional status, employment, occupation, family bonding, and other factors [21].  The authors of this study considered model-based cluster analysis for the following reasons:  Latent Class Analysis (LCA) is an advanced model-based clustering method that uses a probability model and is increasingly being used in social, psychological, and educational research [35–37]. LCA offers a powerful analytical approach for categorizing groups (or ‘‘classes’’) within a heterogenous population and identifies these hidden classes by a set of predefined features. Unlike many other grouping analytical approaches, LCA derives classes using a probabilistic approach[38]. Therefore, instead of analyzing each socio-demographic factor separately, in this study, we have considered a latent class analysis (LCA), which allows for a person-centered approach to understanding how a wide range of socio-demographic factors may cluster together to reflect distinct social classes within a large and heterogeneous population. | Page:6  Lines:21-24  Page:12  Lines:258-263  Page:5  Lines:110-113 |
| 2. | Literature review is rather short. There is huge amount of literature examing the association between childhood socioeconomic environment and later risk of mental disorders using large scale registry and survey data. | Thank you for notifying this issue and suggesting the relevant articles.  We have revised the literature review section and included more background information to justify the study as follows:  “The family structure is a representation of the connection between one's living arrangements, marital status, and biological relatedness[13]”.  “According to the Australian Bureau of Statistics (ABS) report of Australian families with children and adolescents, 89% of families were “intact”, 6% were stepfamilies and 5% were blended families. In addition, 71% of all children lived with both natural parents (bio-logical parents), 4% were in stepfamilies, and 5% were in blended families. This report also stated that the percentage of adolescents living in their household varies with their age. Naturally, children under the age of five were more likely to live with both of their biological parents (78%), followed by those between the ages of 5-9 years (72%). The least likely to be in this scenario (63%) were those between the ages of 15 and 17 years [14].”  “Daundasekara et. al., (2022) used LCA to identify socio-economic and health risk profiles among mothers of young children predicting longitudinal risk of food insecurity [20]”.  ***In addition, according to the suggestions of reviewers 2, the description of the social hierarchy of Australia and the “Diagram of Australian social hierarchy and its characteristics” have been presented in the background section instead of methods section.*** | Page: 4-5  Lines: 93-97  Page:6  Lines: 119-121 |
| 3. | Attrition analysis is missing. | Thank you for raising this issue.  We know that attrition is one of the key methodological concerns in longitudinal or cohort studies. Cross sectional studies evaluate variables at single point in time, therefore, respondents are less likely to drop out once agreed to participate. In cross-sectional survey design, response rate or completion rate is considered to be important for ensuring data quality and statistical precision. The survey data used in the current study achieved a response rate of 55%. Also, the number of cases in this study is 3152, which is much greater than in a nationally representative sample. As a result, we did not examine attrition analysis, although we have noted this in the limitation. |  |
| 4. | Study limitations are completely missing. LCA can produce unstable results, which are difficult to replicate in other datasets. Study design was cross-sectional. Moreover, can reverse causality, child influence parental SES also be possible? Anyhow, presents results don’t have causal interpretation. | Thank you so much for pointing out this issue.  With the strength of the current research, this study has a few limitations. We have included this in our revised manuscript as follows:  **“Strengths and limitations**  The strengths of this study included the use of person-centered approach (i.e., LCA) that concurrently examines the socio-demographic characteristics of children and adolescents and determines the associations between obtained latent classes and their mental and behavioral disorders by using nationally representative YMM data. This study has a few limitations that need to be mentioned. First, the 55% response rate of the survey could have created some bias that weighting did not take into consideration. Second, this study explored the association of the mental and behavioral disorders of children and adolescents with latent classes, but we did not show the relationship between latent classes with various kinds of mental and behavioral disorders in children and adolescents. Moreover, we did not consider the mental health status of the parents/carers may have an impact on the outcome of our study. “Don’t know” responses were omitted in our study, which may have a significant impact on the result. Lastly, the analysis was based on a cross-sectional study and self-reported responses, where causal relationships cannot be established through a cross-sectional study, and self-reported responses compromise the reliability and validity of measurement.” | Pages:23-24  Lines: 481-495 |
| 5. | Very different kind of mental disorders very grouped together. Please conduct sensitivity analyses were different type of mental disorders are analysed separately. In addition, please report prevalence of different type of mental disorders in girls and boys. | Thank you for raising this issue.  “The objectives of our study were to identify the cluster of the items representing socio-demographic characteristics of Australian children and adolescents and then explored the link between the mental and/or behavioral disorders of children and adolescents with class membership and adjusted controlling factors.  Therefore, we have only considered the variable “mental and/or behavioural disorder” in our analysis (whether children and adolescents had any major types of mental and/or behavioral disorders or not), and we did not consider different types of mental disorders separately. This is one of our future objectives and have a plan to write another paper based on this and other objectives.  In addition, according to Shanahan et al. (2013), any particular latent class should not contain less than 5% of the sample [18]. . In that case, performing LCA for different types of mental disorders individually could lead to results that are deceptive and/or fall short of LCA requirements. | Page:7  Lines: 151-154  Page:13  Lines: 277-278 |
| 6. | Discussion is difficult to follow as results are repeated in detail. Please first state your primary results and then discuss them in the context of previous studies. | Thank you for your valuable recommendations. The authors have revised the discussion section according to the reviewer’s suggestion. We hope that the clarity of the section has been improved. | Pages: 21-23 |
| 7. | Potential mechanisms explaining the study findings are currently missing. | Thanks for pointing out this issue.  We have explained how the study findings may be important for policy and practice in the revised version of the manuscript. | Pages:23  Lines: 418-480 |
| Minor points | |  |  |
| 8. | Two decimals should be enough in text and in tables  p-value can’t be 0.000 (table 2) | Thank you for your feedback.  We have considered two decimal points in all our tables 1-6.  P-value has been written as <0.001 instead of 0.000 in the revised manuscript. |  |
| Reviewers# 2 | | | |
|  | Reviewer’s comments | Author’s response |  |
| 1. | Overall, I find the manuscript objective and the methodological approach very interesting. From my point of view, however, the manuscript needs a major revision. In particular, the operationalization of "mental health" should be in accordance with the standards. The authors reported a 12month prevalence of 63.5 % for mental disorders in adolescents. In the manuscript of Lawrence et al. (doi:10.1177/0004867415617836), who used the same data, the 12month prevalence of mental disorders was 13.9 %. | Thanks to the reviewer for providing valuable feedback on the submitted paper.  And thanks again for pointing out this major issues. In a previous study, Irteja et al. (<https://doi.org/10.1371/journal.pone.0231180>) using this YMM data showed that the 12-month prevalence rate of mental disorders of adolescents aged 13-17 years was 34.7%. However, the authors did not clearly explain how they measured mental disorders. In our study, as we include children as young as 11, we investigated at the age range of 11-17 years in our study. While we measured each mental disorder, we created a binary variable and considered the presence of the corresponding mental disorder when at least one of the relevant symptoms was present. Consequently, we then added a variable ‘mental disorder’ in our analysis: whether the adolescent has had any of the following types of mental disorders—anxiety disorders, major depressive disorder, ADHD and conduct disorder. As a result, the prevalence rate was a bit high in our study. However, the distribution of the proportion of mental disorders by various sociodemographic factors in our study showed consistent results with the previous study. For instance, the prevalence of mental disorders was higher in the age group 15 to 17, which was consistent with the findings of Irteja et al. (2020) [43]. Similarly, the prevalence of mental disorders with other socio-demographic factors such as regional status, family blending/ family type, household income, parental education, occupation, employment status, and IRSAD quintile also showed quite similar results with the prior studies.  After considering your recommendation we have deeply reviewed some past studies including Lawrence et al. (2016) to better understand the criteria of the presence of a specific disorder. After that, we have framed the variable “Mental and/or behavioural disorder” as follows:  Several questions in the survey were used to indicate the presence of mental or behavioral problems or distress while not meeting full diagnostic criteria, may be of clinical significance or of concern to parents and carers or children and adolescents. A sub‐threshold level of mental disorder on one or more of the DISC‐IV diagnostic modules, in which symptoms are present but not at a level of severity and/or for a sufficient duration to meet diagnostic criteria. For this analysis, the sub‐threshold level was set at half or more of the required number of symptoms to meet full diagnostic criteria. For example, DSM‐IV criteria for major depressive disorder require the presence of 6 or more symptoms, and DSM-IV/5 criteria for ADHD also requires six (or more) of the associated symptoms. The symptoms that were considered in this study for measuring MDD, for example, were- In the last year, was there a time when CHILD often seemed sad or depressed?, seemed like nothing was fun?, was grouchy or irritable?, lost weight?, lose [his/her] appetite?, gained a lot of weight?, seemed to feel much hungrier than usual?, had trouble sleeping?, slept more during the day than [he/she] usually does?, seemed to do things like walking or talking much more slowly than usual, seemed restless?, seemed to have less energy than [he/she] usually does?, feel tired?, arms legs felt heavy?, blamed for bad things?, nothing do well?, seemed to have trouble keeping [his/her] mind on things?, hard to make decision?, thought about death and died people?, talk seriously about killing?, and take suicide attempt? Similarly, for measuring other mental/behavioural disorders, we have considered the relevant questions about the symptoms of a particular disorders from YMM data dictionary.  We then added a variable ‘mental and/or behavioral disorder’ in our analysis: whether the adolescent has had any of the following types of mental and/or behavioral disorders - major depressive disorder, ADHD, conduct disorder, or anxiety disorders which includes social phobia, separation anxiety disorder, generalized anxiety disorder, and obsessive-compulsive disorder in the past 12 months. Responses included ‘Yes’ (if a child has at least one of these issues, the code is 1) or ‘No’ (coded as 0 if otherwise).  ***After employing this procedure, we have found 12 months prevalence of mental and/or behavioral disorder of children and adolescents aged 11-17 years was 38.5%, whereas Irteja et al. (2020) showed 12-month prevalence rate of mental disorders of adolescents aged 13-17 years was 34.7% [43].*** | Pages:9-10  Lines:  199-223  Page: 14  Line: 310-313 |
| 2. | It would have been easier to comment on the paper if page and line numbers had been added to the text. | Thank you so much for this suggestion.  Page and line numbers have been added to the text. |  |
| 3. | Please proofread your manuscript meticulously and correct the spelling and grammatical mistakes. In parts the wording lacks accuracy | Thank you for your suggestions.  The manuscript has been edited by an English-speaking native, so we hope it now matches the journal standard. |  |
| 4. | Abstract:  Please include the year of data collection in the methods section of the abstract. | Thanks for indicating the issue.  Year of data collection has been included in the methods section of the abstract as follows:  **“Methods:** Children and adolescents aged 11–17 years (n = 3152) were considered from the 2013-2014 Young Minds Matter: The Second Australian Child and Adolescent Survey of Mental Health and Wellbeing”. | Page: 2  Lines: 31-33 |
| 5. | Background:  In the section of the background chapter that begins with “Therefore, instead of analyzing each socio-demographic factor separately, in this study, we have considered a latent class analysis (LCA)…”, I would describe the research gap, not the objective of the study. For example, you could write: Up to date, in these studies, socioeconomic and sociodemographic factors were only analyzed separately, not in combination. | Thank you for this response.  We have written a similar statement three lines before “Therefore, instead of analyzing each socio-demographic factor separately, in this study, we have considered a latent class analysis (LCA)…”,  Now we have re-written this in the following way according to your suggestions.  “To date, in all these studies, those socioeconomic and demographic variables were only analyzed separately not in combination.” | Page: 5  Line: 105-107 |
| 6. | In the last paragraph of the background I would recommend to write:  “Therefore, the FIRST objective of this study is to identify the cluster …”  AND:  “The SECOND objective of this study is to explore the link between adolescent mental health with class membership and adjusted controlling factors.” | Thank you for your advice.  We have revised this section as follows-  “Therefore, the first objective of this study was to identify the cluster of the items representing socio-demographic characteristics of Australian children and adolescents aged 11-17 years by using LCA, and the second objective was to explores the link between the mental and behavioral disorders of children and adolescents with class membership and adjusted controlling factors. | Page:7  Lines: 151-154 |
| 7. | Method:  Please delete the year in brackets of reference 22. | Thank you for pointing out this.  We have removed the year in brackets. | Page: 8  Line: 172 |
| 8. | In the chapter “Measurements” please describe the measurement and operationalization of the sociodemographic variables. The description of the social hierarchy of Australia and the “Diagram of Australian social hierarchy and its‘characteristics” should be presented in the background chapter, not in the methods chapter. Please only provide information related to your study methodology. | Thank you for your nice suggestions.  Measurement and operationalization of the sociodemographic variables were clearly described in the measurement sections as follows:  “Socio-demographic variables used in this study were represented by age (11 - <15 years vs. 15 - <18 years), Sex (male vs. female), income (less than $52,000 as low, $52,000–$129,999 as medium, and more than $130,000 as high), regional status (metropolitan vs. non-metropolitan), primary carer education (bachelor, diploma, and year-12/below), primary carer occupation (employed vs unemployed), index of relative socio-economic advantage and disadvantage (IRSAD) quintile [lowest (most disadvantaged), second, third, fourth and highest (most advantaged)] of the parents, family blending (intact family vs other families) and both parents living status in the household (yes vs no), . The index (IRSAD) summarizes data on the economic and social circumstances of residents and households in a region, including both relative advantage and disadvantage measures. Variable “income” represents the total household income per year before tax (gross income). The variables “family blending” and “both parents living status in the household” indicated the family structure of the corresponding household. Adolescents from non-intact families had a lower perception of family functioning than adolescents from intact families [3]. Families are said to be intact if both biological parents lived there and non-intact families included stepfamily, blended family, lone parent family and other family. In addition, the variable ‘both parents live in the household’ indicates what percentage of children and adolescents lived with both of their parents.”  The description of the social hierarchy of Australia and the “Diagram of Australian social hierarchy and its characteristics” have been presented in the background section.” | Pages: 10-11  Lines: 224-240  Pages: 6-7  Lines:124-142 |
| 9. | Please explain the income variable in more detail. Is the income of the household used? Was the number of household members taken into account (household equivalent income? Is it gross or net income)? | Thank you for your feedback.  In this study, the income variable represents the overall household income per year before tax, which indicates the gross income. | Page:10-11  Lines: 233-234 |
| 10. | What is the difference between “family blending (intact family vs other families)” and “parents’ living status (whether both parents live in the household or not)”? Is it necessary to use both indicators?  I suppose that the two variables measure almost the same thing. Do the variables refer to the biological parents?  I would suggest to use the variable “family type” (“couple family” versus “one-parent family”) as Lawrence et al. (2016) (doi:10.1177/0004867415617836) does. Or alternatively: “original family (yes/no)”.  Furthermore, I am of the opinion that both variables measure family structure and not family functioning. Although it is known that there is a strong association between family structure and family functioning, they are not the same. Therefore, please move the sentence “In 2015, a study found that adolescents from non-intact households had a lower perception of family functioning, father‘s and mother‘s behavioral control, paternal psychological control, and parent-child relational traits than adolescents from intact households [25].” into the discussion. | Thank you for pointing out this.  The variables “Family blending” and “Both parents living status in the household” measured family structure, and both of these variables have a significant impact on family functioning.  In the variable” family blending”, other families include stepfamily, blended family, lone parent family and other family. Moreover, the variable “Both parents living status in the household” means the children and adolescents lived with both of their parents.  According to the ABS (Australian Bureau of Statistics) report of Australian families with children and adolescents (2011), 89% of families were “intact”, 6% were stepfamilies and 5% were blended families. In addition, 71% of all children lived with both natural parents (bio-logical parents), 4% were in stepfamilies, and 5% were in blended families. This report also stated that percentage of adolescents living with their household varied with their age. Naturally, children under the age of five were more likely to live with both of their biological parents (78%), followed by those between the ages of five to nine (72%). The least likely to be in this scenario (63%) were those between the ages of 15 to 17 years. Most of the adolescents in our study also belong to the age group of 15-17 years and a greater percentage of adolescents who were suffering from the mental and behavioral disorders also belongs to this group**.** For that reason, we considered both variables.  Lawrence et.al. (2016), also used the variable “family blending” in their study but they used the name “family type’ and represented as original family, stepfamily, blended family, sole/lone parent family and other family.  We have moved the sentence “In 2015, a study found that adolescents from non-intact households had a lower perception of family functioning, father‘s and mother‘s behavioral control, paternal psychological control, and parent-child relational traits than adolescents from intact households [34]” into the discussion section. | Page: 22  Lines: 458-463 |
| 11. | I would recommend to move the first paragraph of the chapter “Study participants and data analysis” to the end of the chapter “Data source and study design” and rename the chapter "Statistical Analysis". | Thank you for the suggestions.  We have moved the paragraph and change the section name according to your suggestions as-  “Statistical analysis” | Page: 11  Line: 246 |
| 12. | I don’t understand why you adjusted for the variables “inability to pay gas, electricity, or telephone bills on time”, “could not pay the mortgage or rent on time”, “adults/children have gone without meals”, and “households receive a carer benefit or pension in relation to child”. I think it is an overadjustment. You may want to analyze whether the association between class membership and mental disorder is mediated by these variables. But that is not the subject of your study. I recommend to adjust only for household size. | Thanks for raising the issue and valuable suggestions.  According to the reviewers’ suggestions, we have adjusted the variable “household size” only and current adjusted model showed almost similar results as unadjusted model. | Page: 20 |
| 13. | Were weights and survey procedures used in the statistical analysis? Due to the sampling (complex structure of sample), this should be done. | Thank you so much for this feedback.  All the estimates were weighted in the revised manuscript to represent 11-17-year-olds in the Australian population. In particular, the weighting took into consideration the overrepresentation of younger children and households with more than one child between the ages of 4 and 17 as well as the oversampling component of 16- and 17-year-olds. |  |
| 14. | Results:  For the labeling of the age groups, a wrong mathematical symbol was used. It must be “≥15”. Alternatively, you could write “11 to <15” and “15 to <18” (see line 2 of the results chapter and Table 1). | Thank you.  We have changed the label according to your suggestions as follows:  “According to the age group, 51.1% of the total samples were between 15 to <18 years old and the mean age was 14.6 years.” | Page: 14  Lines: 298-299 |
| 15. | “Moreover, the study results showed that 68.1% of parents live in households.” What does this mean? | Thank you so much for querying this.  According to the new analysis, this is 66.1%.  “This indicates that 66.1% of the children and adolescents lived with both of their parents”. | Page: 14  Lines: 302-303 |
| 16. | You reported a 12month prevalence of 63.5 % for mental disorders in adolescents!  In the manuscript of Lawrence et al. (2016) (doi:10.1177/0004867415617836), who used the same data, the 12month prevalence of mental disorders was 13.9 %. I recommend strongly to check the operationalization of the variable. For reasons of comparability, please build the variable analogously to the mentioned reference paper. | Thank you so much for pointing out this major issues.  We have elaborately described this issue in response to the reviewer 2’s, **comment# 1.** |  |
| 17. | The associations between the single sociodemographic variables and mental disorders are not the subject of the study. Therefore, I would delete the section “Distribution of socio-demographic characteristics with adolescents’ mental health” and move Table 2 into the supplement.  After the description of the study sample, I recommend to start with the classes you found. | Thank you for your feedback.  We have arranged manuscript as per your suggestions and placed table 2 in the supplementary information (Table S1). In addition, we started our writing with the classes after the description of the study sample. | Page: 15  Lines: 323-326 |
| 18. | The description of the clusters could be more concise. Please highlight the main characteristics of each class.  The cluster labels of class 3 “Mobile middle class” and class 4 “Growing wealthy class” cannot be deduced from the variables used in the LCA. For example, the most striking criterion of the "growing wealthy class" is that the adolescent does not live with both parents. I recommend to give the classes labels that result from the associations reported in Table 3. | Thank you for your feedback.  After highlighting the main characteristics of each class, we revised the description of the latent classes. Considering your recommendation, we have labelled these classes as follows-  Class-1: Lowest socio-economic status and poor family structure  Class-2: Worst education and occupational attainment and good family structure  Class-3: Average socio-economic status and good family structure.  Class-4: Good socio-economic status and worst family structure  Class-5: Highest socio-economic status and good family structure |  |
| 19. | In Table 4, I suggest to use the recently introduced labels for the classes and to put the class numbers in brackets. | Thank you so much.  We have introduced the labels according to your suggestions in table 3. | Page: 17 |
| 20. | Following the description of the classes, I would like to know the prevalences for mental disorders stratified by class. That is the main result of your study! | Thank you so much for raising this issue. We have shown this on Table 4. Table 4: Distribution of the prevalence of mental and behavioral disorders by class membership Latent classes \| Mental and behavioural disorders \| \| Predicted class membership; \| Yes n(%) \| No n(%) \| n (%); Lowest socio-economic status and poor family structure (class1) \| 330 (52.4) \| 300 (47.6) \| 630 (20.0); Worst education and occupational attainment and good family structure (class 2) \| 148 (35.2) \| 272 (64.8) \| 420 (13.3); Average socio-economic position and good family structure (class 3) \| 320 (32.9) \| 653 (67.1) \| 973 (30.9); Good socio-economic status and worst family structure (class 4) \| 174 (44.1) \| 221 (55.9) \| 395 (12.5); Highest socio-economic status and good family structure (class 5) \| 240 (32.7) \| 494 (67.3) \| 734 (23.3); Total \| 1212 (38.5) \| 1940 (61.5) \| 3152 (100); p-value: <0.001 \| \| \| | Pages: 19-20 |
| 21. | In the section “Regression modeling” I would start with the sentence “Table 5 presents the results of binary logistic regressions …”. The sentences before belong into the method chapter. | Thank you for this important suggestion.  We have revised this part according to your suggestions as follows:  “**Regression modelling**  Table 5 presents the results of binary logistic regressions examining the associations between identified classes and mental and behavioral disorders after adjusting for covariates. Children and adolescents belonging to class 1 (lowest socio-economic status and poor family structure) and class 4 (good socio-economic status and worst family structure) were about 2.26 and 1.62 times more likely to be suffering from mental and/or behavioral disorders compared to their class 5 (highest socio-economic status and good family structure) counterparts (OR [odd ratio]: 2.26, 95% CI [confidence interval]: 1.82-2.82 for class 1, and OR: 1.62, 95% CI: 1.26-2.08 for class 4). Class 2 (worst education and occupational attainment and good family structure) and class 3 (average socio-economic status and good family structure) also showed higher odds of mental and behavioral disorders than did class 5, but they were not statistically significant.”  Note: In the revised manuscript we have changed the reference category for better understanding of the differences among the classes and used class 5 as the reference category. | Pages: 19-20  Lines: 396-406 |
| 22. | Regarding Table 5, 2 decimals suffice for reporting the OR and the 95 % CI. | Thank you. In the revised manuscript we have used two decimals in all the tables |  |
| 23. | The calculation of the OR only allows comparisons to the reference group. If you would calculate predictive margins, you could compare all classes with each other.  Furthermore, when the prevalence of the disease is high, the Prevalence Ratio (PR) would be the better estimator. | Thank you for the feedback. We know that, in case of analysing public health data, odds ratio is commonly used to report the strength of association between exposure and an event. The larger the odds ratio, the more likely the event is to be found with exposure. For better understanding of the of the significant difference among the classes and more precise interpretation, we have changed the reference category in our revised manuscript and used class 5 (Highest socio-economic status and good family structure) as the reference category. As a result, our final study outcome was described as follows: Children and adolescents belonging to the class 1 (Lowest socio-economic status and poor family structure) and class 4 (Good socio-economic status and worst family structure) were about 2.26 and 1.62 times more likely to be suffering from mental and behavioral disorders compared to their class 5 (Highest socio-economic status and good family structure) counterparts”. After considering your recommendations we have determined the predictive margin of the classes. And we found the following results. \| Predictive margin (95% CI); Lowest socio-economic status and poor family structure (class1) \| .52 (0.48-0.56); Worst education and occupational attainment and good family structure (class 2) \| .35 (0.31-0.40); Average socio-economic position and good family structure (class 3) \| .32 (0.29-0.36); Good socio-economic status and worst family structure (class 4) \| .44 (0.39-0.49); Highest socio-economic status and good family structure (class 5) \| .32 (0.29-0.36) This result is similar to the distribution of the prevalence of mental and/or behavioral disorders by classes, which is shown in Table 4. We have interpreted the results in Table 4 as follows: Table 4 shows that the prevalence of mental and/or behavioral disorders of class 1 was 52.4%. This means that, if all the respondents were from class 1, then the average prevalence rate of mental and/or behavioral disorders would have been 0.52. Class 4 shared the lowest predicted class membership (12.5%) but it had a greater prevalence (44.1%) of mental and/or behavioral disorders than did class 2 (35.2%), class 3 (32.9%), and class 5 (32.7%). As a result, it can be deduced that if all respondents had been from class 4, then the prevalence rate of mental and/or behavioral disorders would have been 0.44 on average. In addition, the second highest percentage of adolescents (23.3%) belongs to class 5, only 32.7% of whom suffered from mental and behavioral disorders”. For that reason, we did not present this results in our manuscript. As we have responded in the reviewer 2’s comment# 1 that the prevalence rate of mental and behavioral disorders of children and adolescents aged 11-17 years was found to be 38.5% in our revised manuscript. Therefore, we have only estimated the odds ratio rather than prevalence ratio. | Page: 19  Lines:384-392 |
| 24. | Discussion:  The aim of your study is to analyze the association between the sociodemographic classes and adolescents’ mental disorder and not to analyzed the associations between the single sociodemographic variables and mental disorder. In particular, the results for your approach should be reported and discussed. Please revise the first paragraph of the discussion accordingly. | Thank you for your suggestions. We have revised the first paragraph of the discussion part according to your suggestions as follows:  “In this study LCA was used to identify distinct classes based on various socio-demographic factors of Australian children and adolescents. Patterns of socio-economic and demographic features were characterized by various levels of engagement in social behaviours across multiple domains and are differentially associated with mental and behavioral disorders [15, 22, 34, 43]. Differences in socio-demographic characteristics and mental health issues were observed in the heterogeneous classes, supporting the view that structural factors (e.g., age, gender, region, occupation, education and family blending) influence individuals’ mental health. Using nine distinct socio-demographic factors, we were able to identify 5 latent classes in our study. Mental and behavioral disorders of children and adolescents were mostly prevalent in class 1 and rarely in class 5. Although lowest percentage of children and adolescents were from class 4, prevalence of mental and behavioral disorders in this class was higher compared to class 2, class 3, and class 5. The model of binary logistic regression revealed that, in comparison to class 5, class 1 and class 4 showed higher odds of mental and behavioral disorders even after adjusting for control variable.” | Page: 21  Lines: 419-432 |
| 25. | The limitations of the study are missing completely. | Thank you for pointing out this error. Limitations of the study have been added in the strength and limitation sections in the revised manuscript by the following ways-  **“Strengths and limitations:**  The strengths of this study included the use of person-centered approach (i.e., LCA) that concurrently examines the socio-demographic characteristics of children and adolescents and determined the associations between obtained latent classes and their mental and behavioral disorders by using nationally representative YMM data. This study has a few limitations that need to be mentioned. First, the 55% response rate of the survey could have created some bias that weighing did not take into consideration. Second, this study explored the association of children and adolescents’ mental and behavioral disorders with latent classes, but we did not show the relationship between latent classes with various kinds of mental and behavioral disorders in children and adolescents. Moreover, we did not consider the parents/carers’ mental health status, that may have impact on the outcome of our study. ‘Don’t know responses’ were omitted in our study, which may have a significant impact on the result of the study. Lastly, the analysis was based on a cross-sectional study and self-reported responses, where causal relationships cannot be established through a cross-sectional study, and self-reported responses compromise with the reliability and validity of measurement.” | Pages:23-24  Lines: 481-495 |
| 26. | Neither the discussion nor the conclusions point out the advantage of class analysis over bivariate correlation analysis. | Thank you for your feedback.  We have revised the discussion and conclusion section and highlighted the advantages of latent class analysis over bivariate analysis. |  |
| 27. | References:  Please doublecheck the references. For some references the information is not complete (e.g. reference 24). | Thank you.  We have checked thoroughly and corrected the references. |  |
| Reviewers# 3 | | | |
| 1. | The authors present an interesting analysis with appropriate reference to existing literature.  My major concern about the presented analysis is that the prevalence of mental disorders in this population is very high at 63.5% (Table 2). The participants were a representative sample of 11-17 year olds and based on global prevalences I would have expected the prevalence to be 10-30%. A quick look at national Australian data also suggest that this prevalence is very high. (<https://www.aihw.gov.au/reports/children-youth/australias-children/contents/health/children-mental-illness> ; <https://www.abs.gov.au/statistics/health/mental-health>)  The authors should explain why such a high prevalence was observed e.g. was this population different in some way, and if so then how generalizable are the findings. | Thank you so much for encouraging feedback.  Reviewer 2 also raised the same query.  We have checked and reanalysed our data. In the query number 1 of reviewer 2, we have elaborately described about the measurement of the mental and behavioral disorders.  In the responses to comment 1 of reviewer 2, we also explained why rate was so high in our original submission and what changes we made in the revised version.    After revision, we found the prevalence of mental and behavioral disorders of children and adolescents aged 11-17 years was 38.5% in our study. However, a previous study (Irteja et al. (2020); <https://doi.org/10.1371/journal.pone.0231180.t005>) using the same data showed that the prevalence of mental disorders of adolescents aged 13-17 years was 34.7% |  |
| 2. | Discussion  The authors should include a description of the strengths and weaknesses of the methods and measurements used. For example, some assumptions are made around family functioning dependent on whether the adolescent is living with their parents. There is likely to be evidence in the literature that supports this assumption but in situations where parents are arguing then the family may function better when the parents live in different households. The authors should reflect on the appropriateness of these assumptions. Also, family functioning appears to be measured using just one question around who the adolescent lives with and this warrants some reflection and critique. | Thank you for raising this issue and nice concluding remarks.  We have included strength and limitations in our revised version separately from discussion as follows:  **“Strengths and limitations:**  The strengths of this study included the use of person-centered approach (i.e., LCA) that concurrently examines the socio-demographic characteristics of children and adolescents and determined the associations between obtained latent classes and their mental and behavioral disorders by using nationally representative YMM data. This study has a few limitations that need to be mentioned. First, the 55% response rate of the survey could have created some bias that weighing did not take into consideration. Second, this study explored the association of children and adolescents’ mental and behavioral disorders with latent classes, but we did not show the relationship between latent classes with various kinds of mental and behavioral disorders in children and adolescents. Moreover, we did not consider the parents/carers’ mental health status, that may have impact on the outcome of our study. ‘Don’t know responses’ were omitted in our study, which may have a significant impact on the result of the study. Lastly, the analysis was based on a cross-sectional study and self-reported responses, where causal relationships cannot be established through a cross-sectional study, and self-reported responses compromise with the reliability and validity of measurement”.  However, in our study we have considered two family structure variables (family blending, both parents living status in the household). Both factors have significant impact on family functioning. According to the ABS (Australian Bureau of Statistics) report of Australian families with children and adolescents (2011), 89% of families were “intact”, 6% were stepfamilies and 5% were blended families. In addition, 71% of all children lived with both natural parents (bio-logical parents), 4% were in stepfamilies, and 5% were in blended families. This report also stated that percentage of adolescents living with their household is varies with their age. Naturally, children under the age of five were more likely to live with both of their biological parents (78%), followed by those between the ages of five and nine (72%). The least likely to be in this scenario (63%) were those between the ages of 15-17 years. According to our study higher percentage of children and adolescents aged 15-17 years were suffering from mental and behavioral disorders. | Pages: 23-24  Lines: 481-495 |
| 3. | A main conclusion is that improvement of mental health for children and adolescents requires improved socioeconomic status of the parents and family structure.  The potential importance of the mental health of the parents should be discussed as poor parental mental health could be one of the reasons that the family have poor socioeconomic status and/or why the family structure is disturbed. | Thank you.  According to the reviewers’ suggestions we have revised the conclusions of the abstract as follows.  “Abstract:  **Conclusions:** Among the five latent classes, children and adolescents from classes 1 and 4 have a higher risk of developing mental and behavioral disorders. The findings suggest that the improvement of mental health for children and adolescents requires improving their family structure and the socioeconomic status of the parents.  We have stated in our strength and limitation section that we did not consider parental or carer mental health in this study. | Pages:  3  Lines: 49-52 |
| 4. | Conclusions  Please elaborate on recommendation to screen for patterns of socio-demographic factors to detect and prevent mental disorders. I presume that the authors are not suggesting that mental health or social services should be provided only to certain classes but perhaps they think that some targeting might be possible? | We thank the reviewer for the careful consideration and constructive feedback.  In this study, our target was not only to suggest particular classes for improving mental and behavioural disorders of children and adolescents but also to recommend the corresponding factors that have significant influence on the prevalence of mental and behavioral disorders.  We have revised the conclusions in the following ways:  “The present study identified five classes based on various socio-demographic characteristics and highlighted the association of mental and behavioral disorders of Australian children and adolescents. Class 1 (Lowest socio-economic status and poor family structure) and class 4 (Good socio-economic status and worst family structure) showed higher odds of mental and behavioral disorders among children and adolescents than class 5 (Highest socio-economic status and good family structure). In addition, compared to classes 2, 3, and 5, children and adolescents from class 4 were more likely to have suffered from mental and behavioral disorders, although this class shared the lowest class membership and consisted good socio-economic status. This indicates that, improving the socioeconomic position of the parents as well as the family structures is necessary for improving the mental health of children and adolescents. The associations between the pre-identified clusters and mental health highlighted the importance of screening the patterns of socio-demographic factors as a promising step to detect and prevent mental and behavioral disorders among at-risk children and adolescents more effectively. | Page: 24  Lines:496-509 |
| 5. | Minor comments  Abstract  The sentence ‘However, no study has yet been conducted on a model-based cluster analysis of socio-demographic characteristics with mental health’ could be rephrased to indicate that the authors could not find evidence of such a published study. | Thank you for your recommendations.  We have rephrased this in the following way-  “However, no research has been found on a model-based cluster analysis of socio-demographic characteristics with mental health.” | Page: 2  Lines: 26-27 |
| 6. | It is possible (likely?) that someone has tried this at some point somewhere. | A study has been published in August 2022 (<https://doi.org/10.1371/journal.pone.0272614>) by Daundasekara et al. where the authors used latent class analysis to identify socio-economic and health risk profiles among mothers of young children predicting longitudinal risk of food insecurity [19]. However, that study is not in the Australian contexts, and it is based on US data. Importantly, the scope of our study is distinctly different from that of Daundasekara and colleagues.  We have added this reference in our background section in the revised manuscript as follows:  “Daundasekara et. al., (2022) used LCA to identify socio-economic and health risk profiles among mothers of young children predicting longitudinal risk of food insecurity”. | Page: 6  Lines: 119-121 |
| 7. | Presenting data to 2 decimal points is usually sufficient and easier for the reader to digest. | Thank you for the suggestions.  In the revised version we have used two decimal points. |  |
| 8. | Background  Line 1- are mental health issues rising or is their detection rising? | Thank you for correcting this.  We have corrected this line in the following way-  “Both incidence and prevalence of mental health conditions are rising significantly among young Australians.” | Page: 4  Lines: 81-82 |
| 9. | Measurements  Para 2  ADHD and conduct disorder are behavioural disorders so a more correct description would be ‘mental and behavioural disorders’ | Thank you for indicating this issue.  We have replaced the word “mental disorders” by “mental and behavioural disorders” in the revised version and accordingly we have changed the title of the manuscript as:  **“A latent class analysis of the socio-demographic factors and mental and behavioral disorders of Australian children and adolescents”** |  |
| 10. | Fig 2  The word ‘regions’ should be explained as has not been defined previously. In Table 1 this is defined but then replaced with ‘remoteness’ in Table 4. I recommend the use of consistent terms. | Thank you for pointing out this error-  In the revised version of the manuscript, we have used the label “regional status” in all the tables. | Page: 6  Lines: 126  Table 1, Table 3 |
| 11. | Family blending  Intact families are described as those families in which their parents are married and living together  Other families include step, blended and single-parent families.  If an adolescent is living with both parents but they are not married, which category do they fall into? | Thank you for your feedback.  In this study we have considered both these variables (family blending, and both parents living in the household) as independent family structure variables. Both have significant association with mental and behavioral disorders of children and adolescents.  In the YMM data dictionary, there is no clear indication whether both parents are married or not as we know that non-marital cohabitation is socially and culturally acceptable in the Western World. The question asked- “Both parents living in household?” and the responses was “yes” or “no”. |  |
| 12. | Table 2  Has both row % (mental disorder) and column % (total). This can be a bit confusing for the reader so I suggest you reformat and/or label the % as row and column where appropriate. | Thank you for this suggestion.  We have used the column percentage in the revised version in Table 1 and Table S1. In table 4, we used row percentage in mental and behavioral disorders and column percentage in predicted class membership. |  |
