# Supplementary material for: A latent class analysis of the socio-demographic factors and associations with mental and behavioral disorders among Australian children and adolescents
Source: PLoS One. 2023 May 18;18(5):e0285940. doi: 10.1371/journal.pone.0285940 (PMC10194994; doi:10.1371/journal.pone.0285940)
Supplement: Attachment — Submitted filename: Response_to_Reviewers.docx [file pone.0285940.s004.docx]

April 15, 2023

**A latent class analysis of the socio-demographic factors and associations with mental and behavioral disorders among Australian children and adolescents**

Dear Editor,

Thank you very much for providing us with the editor and reviewer’s feedback and the opportunity to revise the manuscript. We appreciate the editor and the reviewers’ insightful comments and suggestions. We have revised the paper as per the editor and reviewer’s comments and a point-by-point response to the comments is provided below:

Best regards,

Authors

**Reviewer #1 comments and author responses**

Authors have addressed most of the issues I have raised.
I have some minor comments:

**Comment 1:** Literature review is still rather short. The following sentences could be presented in more detail and include more references to previous studies: "Various socio-demographic characteristics such as age, regional status, gender, parental education, parental employment status, and household income can predict mental health and aggressive behaviors in children and adolescents [9–11]. The mental health of children and adolescents is also expressively influenced by their family structure [12]."

***Authors’ responses***

Thank you very much for your comments. We have revised those sentences with more information by presenting them as follows:

According to the Mission Australia’s latest [Youth Survey Report 2022](https://www.missionaustralia.com.au/publications/youth-survey/2618-youth-survey-2022-report/file)*,* 33.9% of young people viewed mental health as a significant national concern. Almost three in 10 (28.8%) young people reported experiencing severe psychological distress, and nearly one-quarter (23.5%) stated feeling lonely most of the time. Little more than half of individuals (53.4%) have at some stage in their lifetimes required mental health care. In 2020, 49.9% of young people were optimistic about their future, but this proportion has steadily decreased since then [7]. **[Page:4; Lines:81-86]**

For instance, household income and parental education had a greater impact on children’s and adolescents’ mental health issues than did parental unemployment or poor occupational status, which refers a low position in the occupational hierarchy [14]. Moreover, children of parents with university degrees are more likely to have greater levels of psychological well-being than children of parents without a university degree [15]. **[Page: 5; Lines: 100-105]**

Numerous studies [20], [21] have shown that children from non-intact families are more likely to experience negative psychological consequences than children from intact families. **[Page: 4; Lines:116-118]**

**Comment 2.** Thank you for including policy and practice recommendations. Potential mechanisms explaining the associations are still missing.

***Authors’ response***

Thank you so much.

The generalized linear model with the log link binomial family (log-binomial regression model) was taken into consideration in the revised manuscript due to the high prevalence of mental and behavioral disorders [48] and determined the prevalence ratio instead of the odds ratio (see Table 5 in the manuscript) [**Page:21]**

**Comment 3:** Potential limitations related to the use of LCA needs to be discussed.

***Authors’ response***

Thank you so much for raising this issue. We have added the limitations related to the use of LCA in the section strength and limitations as follows:

“Third, LCA allocated individuals to classes based on their likelihood of belonging to a class given their patterns of indicator variable scores. However, there is no certainty that the class assignments were completed accurately”. **[Page:24, Lines: 500-503]**

**Reviewer# 2 comments and author responses**

Dear authors,

Thank you for revising the manuscript. The manuscript has now improved significantly. However, I still have a few suggestions which should be addressed.

**Comment 1.** Now a prevalence of mental and behavioral disorders of 38.5% is reported, which is significantly lower than the prevalence in the previous version. However, it is still unclear how mental and behavioral disorder was operationalized and why the prevalence is significantly higher than in other studies. The literature cited does not provide any information on this. This also applies to the study by Irteja et al., which is a self-citation of a co-author. I suppose that you do not consider any functional impairments in your operationalization.
More than a third of the 11- to 17-year-olds are labeled as mentally or behavioral disordered. What is the rationale of operationalizing mental disorders in this way? You refer in your first sentence to the WHO reporting a prevalence of 14% of 10-19-year-olds suffering from mental health difficulties. Lawrence et al. (2016) (https://doi.org/10.1177/00048674156178) reported for Australia a prevalence of 12.8 % in female adolescents and 15.9 % in male adolescents aged 12 to 17.
An important argument for using the standardized operationalization considering functional impairments is that, given this high prevalence, calculating a logistic regression is controversial.
Lines 311-313: The sentence “According to Irteja et al. (2020), 12-months prevalence rate of mental disorders among adolescents aged 13-17 years old was 34.7% [43].” should be moved to the chapters background or discussion.
In Table 4 it is sufficient to report only the prevalence (yes). The other two columns can be deleted.

***Authors’ response***

Thank you for your feedback.

In this study, our outcome variable is “mental and/or behavioral disorders”. We have included children as young as 11 and investigated the age range of 11 to17 years old. Several questions in the survey were used to indicate the presence of mental and/or behavioral problems or distress that despite not meeting full diagnostic criteria, may be of clinical significance, or be of concern to parents, carer givers, or children and adolescents. This study considered all types of anxiety disorders, major depressive disorder, ADHD and conduct disorder as mental and/or behavioral disorders. Each of the mental and/or behavioral diseases was measured using the corresponding criteria, which considered both their major and minor symptoms.

For example, DSM-5 criteria were used to measure conduct disorder (a behavioral problem affecting children and adolescents). If at least four symptoms were present, then its response was “yes”. DSM‐IV criteria for major depressive disorder require the presence of at least six symptoms. Social phobia (SP), separation anxiety disorder (SED), generalized anxiety disorder (GAD), and obsessive-compulsive disorder (OCD) are the types of anxiety disorder (AnxD). All mental and behavioral disorders included in AnxD were measured separately. For example, to measure GAD, anxiety and worry were accompanied by at least three physical or cognitive symptoms. After measuring SP, SED, GAD and OCD, we measured AnxD: whether the children and adolescents have had any of these four types of anxiety disorder. Response included ‘Yes’ and coded as 1 if a child has at least one of these anxiety disorders. ADHD is a **behavior disorder**, usually begins in childhood but may continue into adulthood. It is the most commonly diagnosed behavioral disorder in children. For ADHD, symptoms were divided into two categories: inattention (A1) and hyperactivity (A2). DSM-IV criteria were used to measure A1 and A2 separately. If both criteria A1 and A2 were met, then response was “yes” (Attention-Deficit/Hyperactivity Disorder). In our study we have also considered the response as “yes” for ADHD when criterion A1 was met but criterion A2 was not met; this type of ADHD is referred to as ADHD predominantly inattentive type [1]. Similarly, the response was deemed as “yes” when criterion A2 was met but criterion A1 was not met and it is called ADHD predominantly hyperactive-impulsive type. Attention-Deficit/Hyperactivity Disorder was there after coded as 1 when either or both criteria were met. Finally, we added a variable ‘mental and/or behavioral disorder’. If a child has at least one of the mental or behavioral issues, the responses was “Yes”.

The prevalence of mental or behavioral disorders is comparatively higher in our study because we accounted for all the major and minor symptoms associated with each of them. In addition, we have considered the partial requirements for confirming the presence of the corresponding behavioral problems (such as ADHD). The main reason for doing that was to determine the class-wise prevalence of mental and behavioral disorders in children and adolescents based on their initial symptoms in order to facilitate early diagnosis. According to our study objectives, we intended to investigate the relationship between mental and behavioral disorders of children and adolescents and various latent classes in order to distinguish vulnerable classes from privileged classes. As is well known, some mental and/or behavioral disorders are the most frequently diagnosed disorders in children and adolescents, therefore, it is important to ascertain their prevalence by class at the earliest possible stage in order to facilitate early diagnosis and treatment. This might help reduce a country's annual health care cost burden.

According to previous studies (e.g., Lawrence et al. 2015), the prevalence of mental disorders is quite low because they did not consider partial diagnostic criteria to confirm the presence of corresponding disorder. However, we believe that the prevalence of mental disorder is actually much lower, although the Mission Australia’s new [**Youth Survey Report 2022**](https://www.missionaustralia.com.au/publications/youth-survey/2618-youth-survey-2022-report/file) revealed an alarming information about the mental health of young Australian. According to this report, 33.9% of young people believed mental health to be a significant national concern. Almost three in 10 (28.8%) young people reported experiencing severe psychological distress, and nearly one in four (23.5%) said they felt lonely most of the time. About half (53.4%) have at some point in their lives needed assistance with their mental health. While half (49.9%) of young people were positive about their futures, this percentage has increasingly declined since 2020. **[Page:4; Lines:81-86]**

We estimated the prevalence ratio using the log link binomial family in the generalized linear model (log binomial regression model), as per your recommendation and the reference [48] due to the high prevalence of mental and behavioral disorder, rather than the odds ratio of logistic regression. **[Page:21]**

**Table 5** Log-binomial regression model of predicting latent class membership (unadjusted and adjusted) and control variables.

| **Latent classes (ref.** Highest socio-economic status and intact family structure (Class 5)) | Unadjusted | | Adjusted^a^ | |
| --- | --- | --- | --- | --- |
|  | Coefficient | PR  (95% CI of PR) | Coefficient | PR  (95% CI of PR) |
| Good socio-economic status and non-intact family structure (Class 4) | 0.30^***^ | 1.35  (1.16, 1.57) | 0.29^***^ | 1.35  (1.15, 1.56) |
| Average socio-economic status and intact family structure (Class 3) | 0.01 | 1.01  (0.87, 1.15) | 0.01 | 1.01  (0.88, 1.15) |
| Worst education and occupational attainment and intact family structure (Class 2) | 0.07 | 1.08  (0.91, 1.27) | 0.08 | 1.09  (0.92, 1.28) |
| Lowest socio-economic status and non-intact family structure (Class 1) | 0.47^***^ | 1.60  (1.41, 1.82) | 0.47^***^ | 1.60  (1.41, 1.82) |
| Note: PR-Prevalence ratio; CI-Confidence interval; *: p<0.05; **: p<0.01; ***: p<0.001  ^a^ Control variable: “Household size”. | | | | |

**Comment 2:** Lines 311-313: The sentence “According to Irteja et al. (2020), 12-months prevalence rate of mental disorders among adolescents aged 13-17 years old was 34.7% [43].” should be moved to the chapters background or discussion.
In Table 4 it is sufficient to report only the prevalence (yes). The other two columns can be deleted.

***Authors’ response***

According to the reviewer’s suggestions, the sentence “According to Irteja et al. (2020), 12-months prevalence rate of mental disorders among adolescents aged 13-17 years old was 34.7% [10] has been moved to the background section. **[Page:4; Lines:89-91]**

In addition, reporting only the prevalence other two columns have been deleted from Table 4. **(Page:20)**

**Comment 3:** The operationalization of the variables “family blending” and “both parents living status in the household” is still not clearly described in the method chapter (lines 234, 235). It is also still unclear what’s the difference between both variables.
From my point of view, both family structure variables are very similar, so that the family structure has the double weight in the LCA. This in turn leads to the strong division of the classes regarding the family structure.
Additionally, the description of family types that do not consist of both biological parents is stigmatizing. The family structure should not be labeled as “good”, “bad” or “worst”. You could describe the family structure in a more objective way, e.g.: "The cluster is characterized by a very high proportion of non-intact families." or: “Class 4 - High socioeconomic status and non-intact family structure”.
Furthermore, the aim of health promotion and prevention is not to improve the family structure (lines 51,52 and 505), but to improve the mental health of children and adolescents living in non-intact families. From my point of view, the stigmatizing description regarding some family structures needs to be revised throughout the manuscript.
And please move the sentence “Adolescents from non-intact families had a lower perception of family functioning than did adolescents from intact families [34].” (lines 235-237) from the method chapter into the chapters background or discussion.
Lines 95-99: What is about the single-parent families? Are they part of the intact families?
Lines 302-303: How can it be that 66.1% live with both parents, but only 61.8% are considered to be living in an intact family?
Line 450: Non-intact family structures do not “consequently” possess weak family functioning. But: Referring to other studies, it can be assumed that a non-intact family structure is associated with a weak family function.

***Authors’ responses:***

***Thank you for your comments and suggestions.***

In this study, the variable “family blending” contained the categories intact family and other families (stepfamily, blended family, and lone parent family). Families are said to be intact if both biological parents lived together, and according to ABS [18], an intact family is a couple family with at least one child who is the natural or adopted child of both partners in the couple, and no step children.

The variable “Both parents living status in the household” in our study refers to the children and adolescents who reside with both parents. However, in the YMM data dictionary there is no clear indication of whether or not both parents were biological. Others couple families, such as blended or step couple, may be included here. For whole sample (n=6310) in the YMM data (parent), we have found that 73.8% children and adolescents aged 4 to 17 years lived with both parents and 68.6% belonged to intact families. In our study, about 66.1% of children and adolescents aged 11-17 years lived with both parents and 61.3% were members of intact families. We included the variable "both parents living status in the household" as a family structure variable in our study to examine the variation in mental health issues among adolescents who lived with both parents versus those who do not, where both parents belong to any form of couple families (including step-family and blended family). The family structure variable “family blending” was also used in this study, where adolescents were either from intact families or non-intact families. Intact families refer to families in which both biological parents are present in the household. Both variables contribute independently to the formation of a healthy family structure. For that reason, we have included both variables in our study. Consequently, we believe it is impractical to double the weight in the LCA while forming clusters. This is evidenced by the similarity of the clustering patterns when one variable is removed.

According to the reviewers’ suggestions, we have renamed the classes as follows:

Class-1: Lowest socio-economic status and non-intact family structure

Class-2: Worst education and occupational attainment and intact family structure

Class-3: Average socio-economic status and intact family structure.

Class-4: Good socio-economic status and non-intact family structure

Class-5: Highest socio-economic status and intact family structure

We have revised the description regarding family structure throughout the manuscript.

**#** As per your suggestion, we have moved the sentence “Adolescents from non-intact families had a lower perception of family functioning than did adolescents from intact families [19]” from method section to the background section. **[Page:5; Lines:115-116]**

**#** In YMM data, single-parent families are the part of non-intact families.

**#** In an intact family, parents should be biological. However, in the variable “both parents living status in the household”, all couple family’s parents (may or may not biological parents) were included here. It might be the reason that 66.1% of children and adolescents lived with both parents, however, only 61.8% were from intact families.

**#** In our previous manuscript we have written that classes 1 and 4 have very poor family structures and consequently possess weak family functioning. According to past research, good family functioning has a positive impact on the mental health of children and adolescents.

In the revised manuscript, we have written this as follows:

“Children and adolescents in classes 1 and 4 have non-intact family structures and consequently possess weak family functioning. According to past research, good family functioning has a positive impact on the mental health of children and adolescents.” **[Pages:22-23; Lines: 459-461]**

**Comment 4:** I would suggest changing the manuscript title as follows: A latent class analysis of the socio-demographic factors and associations with mental and behavioral disorders among Australian children and adolescents.

***Authors’ response***

According to your suggestion we have changed the manuscript title as follows:

“A latent class analysis of the socio-demographic factors and associations with mental and behavioral disorders among Australian children and adolescents”

…………………………….

Additional reference:

[1]	S. A. and M. H. S. A. SAMSHA, “DSM-5 Changes: Implications for Child Serious Emotional Disturbance (unpublished internal documentation),” no. June, p. 66, 2016, [Online]. Available: www.ncbi.nlm.nih.gov/books/NBK519712/table/ch3.t8/
