# Supplementary material for: A latent class analysis of the socio-demographic factors and associations with mental and behavioral disorders among Australian children and adolescents
Source: PLoS One. 2023 May 18;18(5):e0285940. doi: 10.1371/journal.pone.0285940 (PMC10194994; doi:10.1371/journal.pone.0285940)
Supplement: Attachment — Submitted filename: Response_to_Reviewers.docx [file pone.0285940.s005.docx]

May 4, 2023

**A latent class analysis of the socio-demographic factors and associations with mental and behavioral disorders among Australian children and adolescents**

Dear Editor,

Thank you very much for providing us with the academic editor and reviewer’s feedback and the opportunity to revise the manuscript. We appreciate the editor and the reviewers’ insightful comments and suggestions. We have revised the paper as per the editor and reviewer’s comments and a point-by-point response to the comments is provided below:

Best regards,

Authors

**Reviewer #1 comments and author responses**

**Comment:** Thanks for addressing all the issues. I have no further comments. I recommend manuscript to be accepted for publication in Plos One.

***Authors’ responses***

Thank you very much for your comments.

**Reviewer #2 comments and author responses**

**Comment 1:** Dear authors, Thank you for revising the manuscript a second time. The manuscript has now improved again significantly.

However, I still have concerns about the operationalization of the variable "mental and behavioral disorders" and a resulting overdiagnosis of mental and behavioral disorders among adolescents. Of course, in terms of freedom of research, you can operationalize "mental and behavioral disorders" as you see fit. In my opinion the operationalization as well as the problem of overdiagnosis should be discussed critically in the limitation section.
Therefore, I would like to ask you to include the criticism regarding an overdiagnosis of mental and behavioral disorders among adolescents as well as the reasoning from your answer letter (see text below) to the limitations chapter in the manuscript (perhaps slightly shortened or adapted). Then – despite my concerns – I agree with the operationalization of the variable.
-> The part from your answer letter: “The prevalence of mental or behavioral disorders is comparatively higher in our study because we accounted for all the major and minor symptoms associated with each of them. In addition, we have considered the partial requirements for confirming the presence of the corresponding behavioral problems (such as ADHD). The main reason for doing that was to determine the class-wise prevalence of mental and behavioral disorders in children and adolescents based on their initial symptoms in order to facilitate early diagnosis. According to our study objectives, we intended to investigate the relationship between mental and behavioral disorders of children and adolescents and various latent classes in order to distinguish vulnerable classes from privileged classes. As is well known, some mental and/or behavioral disorders are the most frequently diagnosed disorders in children and adolescents, therefore, it is important to ascertain their prevalence by class at the earliest possible stage in order to facilitate early diagnosis and treatment.”

***Authors’ responses***

Thank you for your comment.

We have included the following criticism regarding the overdiagnosis of mental and behavioral disorders in adolescents, as well as the reasoning in the strength and limitation sections:

“Second, the prevalence of mental or behavioral disorders is comparatively higher in our study because we considered the partial criteria for confirming the existence of specific mental and/or behavioral problems (such as ADHD) by taking into consideration all of their symptoms. The main reason for doing that was to determine the class-wise prevalence of mental and/or behavioral disorders in children and adolescents based on their initial symptoms to facilitate early diagnosis. According to our study objectives, we intended to investigate the relationship between mental and behavioral disorders of children and adolescents and various latent classes in order to distinguish vulnerable classes from privileged classes. As is widely known, some mental and/or behavioral disorders are the most commonly diagnosed disorders in children and adolescents, therefore, it is important to ascertain their prevalence by class at the earliest possible stage to facilitate early diagnosis and treatment.” However, over-diagnosing mental issues might lead to mis-medication and social stigmas.” **[page:24; lines: 495-507]**

**Comment 2:**  Secondly, regarding the family structure, I still find your wording partially imprecise or even discriminating.
Lines 53-55 (in the clear copy version): “The findings suggest that the improvement of mental health for children and adolescents requires improving their family structure and the socioeconomic status of the parents.”
I suggest rephrasing the sentence as follows:
-> The findings suggest that health promotion and prevention as well as combating poverty are needed to improve the mental health in particular among children and adolescents living in non-intact families and in families with a low socio-economic status.

Please also rephrase the sentence in lines 482-484 and 517-518, accordingly. I added a suggestion at the end.

***Authors’ responses:***

Thank you so much for your suggestions.

We have revised the conclusion of the abstract as per your suggestions as follows:

“Conclusions: Among the five latent classes, children and adolescents from classes 1 and 4 have a higher risk of developing mental and behavioral disorders. The findings suggest that health promotion and prevention as well as combating poverty are needed to improve the mental health in particular among children and adolescents living in non-intact families and in families with a low socio-economic status.” **[page: 03; lines: 53-56]**

We rephrased the sentences in the mentioned lines (482-484) in discussion and (517-519) in conclusions, accordingly as per your recommendations as follows.

“To enhance mental health, particularly among children and adolescents living in non-intact families and in households with low socioeconomic status, health promotion and prevention as well as tackling poverty are necessary.” **[pages:23-24; lines:485-488]**

“The findings indicate that health promotion and prevention as well as combating poverty are needed to improve mental health among children and adolescents living in non-intact families and in families with a low socio-economic status.” **[pages:25; lines:524-526]**

**Comment 3:** In the methods chapter, you refer to ASB's definition that single-parent families are not considered to be intact families. Therefore, please check the following sentence again and consider the single-parent families:
Lines 108-110: "According to the Australian Bureau of Statistics (ABS) report (2013) on Australian families with children and adolescents, 89% of families were “intact”, 6% were stepfamilies and 5% were blended families [18]."

**Authors’ responses:**

Thank you for your comment.

Lines 108-110 contain information regarding couple families only and we presented this according to the ABS report.

However, according to your suggestions, we have revised the mentioned lines after including the information regarding single-parent family according to ABS report as follows:

“According to the Australian Bureau of Statistics (ABS) report (2013) on Australian families with children and adolescents, the majority of children (63–78%) in all age groups lived with both of their biological parents, 15-25% lived in a single-parent family, only 5–6% lived in a blended family, and 2–7% lived in a stepfamily.” **[page:05; lines:109-112 ]**

**Comment 4:** Lines 455-457: "The main disadvantage of this class was that no children and adolescents had come from intact families, and no one lived with either of their parents, thereby indicating relatively poor family functioning."
I suggest rephrasing the sentence as follows:
-> The main disadvantage of this class was that no children and adolescents had come from intact families, and no one lived with both parents, thereby indicating relatively poor family functioning.

Authors response:

Thank you so much. We have rephrased this sentence as per your suggestions**:**

“The main disadvantage of this class was that no children and adolescents had come from intact families, and no one lived with both parents, thereby indicating relatively poor family functioning.” **[page:22 lines:458-460]**

**Comment 5:** Lines 459-461: "In our study, we found that children and adolescents in classes 1 and 4 have very poor non-intact family structures and consequently possess weak family functioning."
I suggest rephrasing the sentence as follows:
-> In our study, we found that most children and adolescents in classes 1 and 4 lived in non-intact families which is more often associated with poorer family functioning.

**Authors’ responses:** Thank you so much. We have rephrased this sentence as per your suggestions. **[page:22-23; lines:462-464]**

**Comment 6:** Could you please clarify this sentence? I don't understand what is meant here.
Lines 473-475: "The ABS report on Australian families with children and adolescents (2011) showed that the lowest percentage of adolescents 15–17 years old lived with both of their natural parents [13]."

**Authors’ responses: Thank you for your comment.**

Lines 473-475 meaning: According to ABS report, children under the age of five years were the most likely to live with both biological parents (78%), followed by those aged five to nine (72%). Those between the ages of 15 and 17 were the least likely to be in this situation (63%).

To emphasize the value of family structure, we brought this up (lines 473-475) in the discussion part in our study.

**Comment 7:** With regard to the conclusions, I suggest not to re-present the results again but to move the sentences from lines 482-490 into this chapter.
Suggestion for the conclusions chapter:
-> "The present study has some important public health implications as it has identified the cluster of items that represent socio-demographic characteristics and revealed their associations with the mental and behavioral disorders of children and adolescents. The significant impact of individual class on the mental and behavioral disorders will help government and non-government agencies as well as practitioners differentiate the vulnerable classes from privileged classes in formulating health-related policy and strategy." The findings indicate that health promotion and prevention as well as combating poverty are needed to improve the mental health in particular among children and adolescents living in non-intact families and in families with a low socio-economic status.

**Authors’ responses:**

Thank you for your suggestions.

We have moved the mentioned lines from discussion part to conclusion part and revised the conclusion as follows:

“The present study has some important public health implications as it has identified the cluster of items that represent socio-demographic characteristics and revealed their associations with the mental and behavioral disorders of children and adolescents. The significant impact of individual class on the mental and behavioral disorders will help government and non-government agencies as well as practitioners differentiate the vulnerable classes from privileged classes in formulating health-related policy and strategy. The findings indicate that health promotion and prevention as well as combating poverty are needed to improve mental health among children and adolescents living in non-intact families and in families with a low socio-economic status.” **[page:25; lines:519-526]**

…………………..
